# Interstitial glucose during sanctioned competition and during high‐intensity training in highly trained collegiate male endurance athletes: A real‐world continuous glucose monitoring study

**DOI:** 10.14814/phy2.71099

**Published:** 2026-09-09

**Authors:** Taira Kajisa, Toshiyuki Sakai

**Affiliations:** ^1^ Graduate School of Interdisciplinary New Science Toyo University Kawagoe Saitama Japan; ^2^ SympaFit Inc. Bunkyo‐ku Tokyo Japan; ^3^ Department of Student, Student Support Division Toyo University Kawagoe Saitama Japan

**Keywords:** continuous glucose monitoring, field physiology, hyperglycemia, nutrition strategy, performance monitoring, time‐series analysis

## Abstract

Real‐world evidence on glucose behavior during competition in highly trained athletes is scarce. We characterized interstitial glucose dynamics in collegiate endurance athletes during sanctioned competitions using continuous glucose monitoring (CGM), with within‐subject comparisons to high‐intensity training performed at the same prescribed race pace. Twenty male runners and race walkers (Tier 3: Highly Trained/National) wore CGM during 36 races, contributing 20 paired race‐versus‐training comparisons. Glucose rose from baseline to 8.3 ± 1.5 mM 1 h pre‐race, peaked at 11.2 ± 2.2 mM during competition (mean difference + 3.12 mM, 95% CI +2.72 to +3.53; Cohen's dz. = 1.45), and declined to 6.7 ± 1.7 mM post‐race (*F*(2,220) = 225.79, *p* < 0.001, partial *η* ^2^ = 0.67). Within‐subject maximum glucose was higher during competition than during training (12.91 ± 1.93 vs. 11.02 ± 1.18 mM; mean difference + 1.89 mM, 95% CI 1.06–2.72; Cohen's dz. = 1.07), with a geometric mean ratio of 1.166 (95% CI 1.088–1.249), approximately 17% higher peaks in competition. These findings describe a reproducible pattern of higher peak glucose in competition than in training matched on the recorded external characteristics. Internal exercise load was not measured, so the difference cannot be attributed to the competitive context rather than to differing internal exercise intensity.

## Graphical abstract

Continuous glucose monitoring in highly trained athletes shows competition glucose peaks ~17% higher than in training performed at the same prescribed race pace. Internal exercise load was not measured.

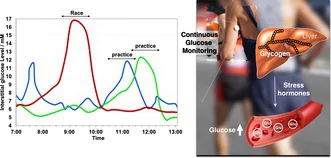

## 1 INTRODUCTION

Athletes depend on optimal physical and mental conditioning to perform at their peak during crucial competitions (Pandian et al., 2022). Elite sports performance occurs under substantial psychological pressure, requiring the integration of multiple physiological systems, and understanding these competition‐specific responses is crucial for optimizing performance at critical moments. During extended exercise, athletes rely on a combination of carbohydrate metabolism, lipid metabolism, and amino acid metabolism to maintain energy supply (Hargreaves & Spriet, 2020; Tipton & Wolfe, 2004). Glucose serves as a primary fuel source for high‐intensity efforts, with highly trained endurance athletes carefully managing their carbohydrate intake before and during competitions to optimize performance (Hargreaves & Spriet, 2020; Thomas, Erdman, & Burke, 2016).

From a physiological perspective, the stress of competition can trigger sympathetic activation and catecholamine release, which in turn elevates hepatic glucose output and alters circulating glucose availability (Hems & Whitton, 1980; Schmidt & Weinshenker, 2014; Sherwin & Sacca, 1984). In adrenalectomised humans, exogenous adrenaline infusion during exercise directly increases hepatic glucose production, providing causal evidence that adrenergic signaling is a primary driver of exercise hyperglycemia (Howlett et al., 1999). Field evidence of stress‐induced hyperglycemia under acute arousal further supports this pathway (Kruyt et al., 2012). The “fight or flight” response activated during competitive stress increases catecholamine release, primarily epinephrine and norepinephrine, which can influence glucose availability through multiple pathways (Kruyt et al., 2012; Qiao et al., 2017; Rui, 2014; Sherwin & Sacca, 1984). Competition‐related arousal may also trigger cortisol release, further promoting glucose production through gluconeogenesis and glycogenolysis (Hems et al., 1978). These stress‐induced hormonal responses can potentially create metabolic conditions during competitions that differ markedly from training environments, even when exercise intensity is matched (Hems & Whitton, 1980).

The present study focuses on the descriptive characterization of glucose patterns using continuous glucose monitoring (CGM) as a practical, non‐invasive field monitoring tool. While we observe glucose patterns that are consistent with stress‐mediated hepatic glucose mobilization, we interpret these findings cautiously and discuss them in the context of established stress physiology rather than claiming definitive causation. Although we recognize that definitive mechanistic attribution would require controlled experimental manipulation of psychological stress, direct hormonal measurements, and standardized course conditions, our primary objective is to document real‐world glucose patterns in the competitive environment where athletes' performance is most critical. This approach prioritizes ecological validity and provides immediately applicable insights for sports practice, while acknowledging the trade‐offs inherent in field versus laboratory research paradigms.

Some athletes experience what is colloquially termed an “adrenaline rush”– moments when performance temporarily exceeds typical capabilities (Kruyt et al., 2012; Schmidt & Weinshenker, 2014). Conversely, others may underperform due to suboptimal regulation of these same physiological responses. The balance between beneficial arousal and detrimental stress likely influences glucose regulation and subsequent energy availability during competitions.

Current knowledge of glucose fluctuations during exercise comes primarily from controlled laboratory settings or training environments (Hill et al., 2018; Ishihara et al., 2020; Suzuki et al., 2015; Thomas, Pretty, et al., 2016). Research involving soccer players found that 70% performed in a hyperglycemic state (> = 6 mM) during matches, with interstitial glucose elevations sometimes reaching 10 mM (Thomas, Erdman, & Burke, 2016). Similarly, studies of long‐distance runners have shown interstitial glucose concentration increases up to 10 mM during training runs (Suzuki et al., 2015). However, these investigations typically occur in relatively controlled environments that lack the psychological intensity of major competitions.

Despite prior advancements, little is known about glucose responses under the psychological stress of real competitions where performance outcomes are critical. The role of stress‐induced glucose regulation remains underexplored, despite its potential relevance for athlete performance and preparation (Hems et al., 1978; Kruyt et al., 2012; Ramnanan et al., 2011; Sherwin & Sacca, 1984). Recent innovations in CGM enable real‐time glucose tracking during actual events, providing fine‐grained, non‐invasive data on glucose trends across competition phases (Bowler et al., 2023; Hill et al., 2018; Ishihara et al., 2020; Suzuki et al., 2015; Thomas, Pretty, et al., 2016). This study used CGM to examine glucose patterns in highly trained/national athletes during actual competitions. We asked whether glucose responses during competition differ from those during training performed at the same prescribed pace. By analyzing long‐distance race data, we aimed to characterize competition‐specific glucose dynamics and offer evidence‐based insights to guide personalized nutrition and stress management strategies in sports performance.

## 2 METHODS

### 2.1 Preliminary methodological validation of SMBG–CGM feasibility during high‐intensity running under fasted conditions

To evaluate correlation between blood and interstitial glucose during exercise, a sub‐elite male runner (40s, HbA1c 5.1%, fasting glucose 5.4 mM) conducted parallel self‐monitoring of blood glucose (SMBG) and CGM measurements. The sub‐elite runner was defined by performance with a marathon personal best <3 h. The concordance between interstitial glucose measured by CGM and capillary blood glucose at rest and in daily living is well established (Rebrin & Steil, 2000; Zaharieva et al., 2019); CGM‐capillary concordance during exercise has been further characterized (Coates et al., 2023; Ranahan et al., 2025). Blood glucose data were collected by SMBG using Glutest Neo Alpha®, which is an enzymatic glucose sensor with finger prick using a Gentlet® (Sanwa Kagaku Kenkyusho Co. Ltd., Nagoya, Japan). Interstitial fluid glucose concentrations were monitored using FreeStyle Libre 2® flash glucose monitoring systems (FreeStyle Libre system; Abbott Diabetes Care, Alameda, CA, USA). We monitored the blood glucose levels of a sub‐elite runner who fasted for 12 h starting at 19:00 the previous evening. The runner, equipped with a CGM, ran a distance of 5 km. Blood glucose levels were also measured at regular intervals using SMBG. To align with the CGM's 15‐min averaging cadence, SMBG measurements were obtained at 15–30‐min intervals throughout the protocol. This athlete completed an oral glucose tolerance test (OGTT) after the high‐intensity running. As a definition of high‐intensity, we referred to a pace faster than the T pace, which is approximately 85% of maximum heart rate and 80% of maximum oxygen uptake (VO2max), based on the Karvonen method and the Jack Daniels Running Formula (Daniels, 2013; Davis & Converti, 1975). This single‐participant test was conducted for technical validation only, not for generalizable physiological inferences.

### 2.2 Long‐term CGM monitoring across a full marathon race under nonfasted conditions

For long‐term monitoring, the CGM was worn for 18 consecutive days including race day.

### 2.3 Main study: Highly trained runners during sanctioned competitions

In main experiment, CGM data were collected for 20 members of highly trained runners (male, early 20's) belonging to track and field in university, who specialize in long‐distance running or race walking, and participated in track, road race, and relay competitions while equipped with CGM. Athletes classified Tier 3: Highly Trained/National (McKay et al., 2022). All athletes adhered to a structured lifestyle within a dormitory setting, with regulated sleep and wake times. Nutritional intake was consistent across participants, with the same dormitory‐provided meals of standardized staple‐carbohydrate content; the same dietary regimen applied on competition and training days, although individual food‐item diaries were not recorded. Training was conducted 6 days per week, and body‐fat percentage was generally maintained in the 5%–15% range (absolute body‐fat percentage points). The cohort contributed 36 CGM datasets from sanctioned race events; because some athletes were monitored in more than one race, the total number of race‐event observations exceeds the number of unique athletes. Analyses that required within‐subject structure treated participant as the repeated factor where appropriate. All races were completed in the nonfasted state under athletes' usual pre‐race routines. As part of team routine, athletes completed solid carbohydrate intake by > = 120 min before race start to minimize acute exogenous effects on interstitial glucose during competition. During races, carbohydrate‐containing fluid intake was prohibited under event regulations; therefore, in‐race carbohydrate drink intake did not confound race‐phase glucose profiles. No carbohydrate was ingested during the matched high‐intensity training bouts, paralleling the in‐race condition; thus during‐exercise feeding did not differ between conditions. Critically, 15 of 20 athletes also wore CGM sensors during high‐intensity training sessions performed at the same prescribed race pace, providing within‐subject paired comparison data between competition and training conditions. Pairs were matched by course type. Track pairs used the same 400‐m track. Road pairs used habitual training routes with similar expected elevation; detailed elevation metrics were not consistently available. Both the competition and the matched training were executed at the same prescribed race pace (R pace). Prescribed pace constrains external mechanical work; it does not establish that internal physiological load was equivalent between the two conditions. Internal load was not measured: heart rate, power, and blood lactate were not recorded, and the pace actually achieved in each session was not documented. The external characteristics of each of the 20 competition‐training pairs are given in Table 3. Athletes satisfied the entry criteria for intercollegiate competitions as well as top long‐distance races in Japan. The selection was made from athletes who had been chosen as the top performers within their respective teams; athletes were recruited from a single university track‐and‐field team, and all eligible top performers entered in sanctioned intercollegiate competitions were invited to participate and provided written informed consent. Details regarding the athletic events included are shown in Table 1. We included only race events in which athletes maintained “R pace”—defined according to Daniels' Running Formula as race‐pace intensity derived from current performance (VDOT) tables—so that the analyzed efforts reflected high absolute intensity (Daniels, 2013). All procedures were conducted in accordance with the Declaration of Helsinki and were approved by the Ethics Review Committee for Medical Research Involving Human Subjects at Toyo University (TU2021‐006). Data collection was carried out for all athletes after explaining the experimental methods and associated risks and obtaining written informed consent through signed consent forms.

**TABLE 1:** Collected data information on athletes in actual races and their type of competition.

Sample number	Type of competition	Race distance (km)
*n* = 2	Run, track	10
*n* = 6	Run, road (relay)	5.8, 6.2, 6.4, 8.0, 8.5, 10.2
*n* = 8	Run, road (relay)	9.5, 11.1, 11.8, 11.9, 12.4, 12.8, 17.6, 19.7
n = 8	Run, road (relay)	20.8, 20.9, 21.3, 21.4, 23.0, 23.1
*n* = 1	Run, cross country	10
*n* = 2	Race walk, road	20, 35
*n* = 3	Run, track	10
*n* = 1	Race walk, road	10
*n* = 2	Run, road	21.0
*n* = 3	Run, track	5
*n* = 36	16,360 plots from 3 days before the race day to 3 days after the race day	

*Note*: Some athletes contributed more than one race observation; total observations = 36 across 20 athletes. Race distances listed for relay events are the individual relay legs run by each athlete. Shorter events were performed at higher relative intensity and longer events at lower intensity; nevertheless, the within‐subject competition‐training contrast (which holds pace constant) was consistent across distances.

### 2.4 Statistical analysis

CGM data across events were statistically analyzed. In the sub‐elite runner, glucose variability data were plotted for 18 days. The 36 glucose‐variability datasets for highly trained runners were plotted for 1 week, 3 days before and after each race day. Statistical analyses were performed using Microsoft Excel 2016 (Microsoft Corp., Redmond, WA, USA). We compared glucose levels before, during, and after races using a one‐way analysis of variance (ANOVA), and calculated summary statistics for glucose levels at each timepoint (mean, median, standard deviation, interquartile range). Paired, two‐sided *t*‐tests evaluated three prespecified within‐subject contrasts (pre −60 min vs. during; during vs. +60 min; pre −60 min vs. +60 min). To control the family‐wise error rate at alpha = 0.05, we applied the Holm‐Bonferroni step‐down procedure within this contrast family. We report mean differences with 95% confidence intervals and within‐subject effect sizes (Cohen's dz.; Hedges' g with small‐sample correction). A one‐way repeated‐measures ANOVA (time: pre, during, post) tested the omnibus effect; when sphericity was violated, Greenhouse–Geisser‐corrected degrees of freedom and p‐values were reported. Partial η^2^ is provided as the ANOVA effect‐size index.

## 3 RESULTS

### 3.1 Preliminary study: Estimation of glucose fluctuations during high‐intensity running of a sub‐elite runner

**FIGURE 1:**
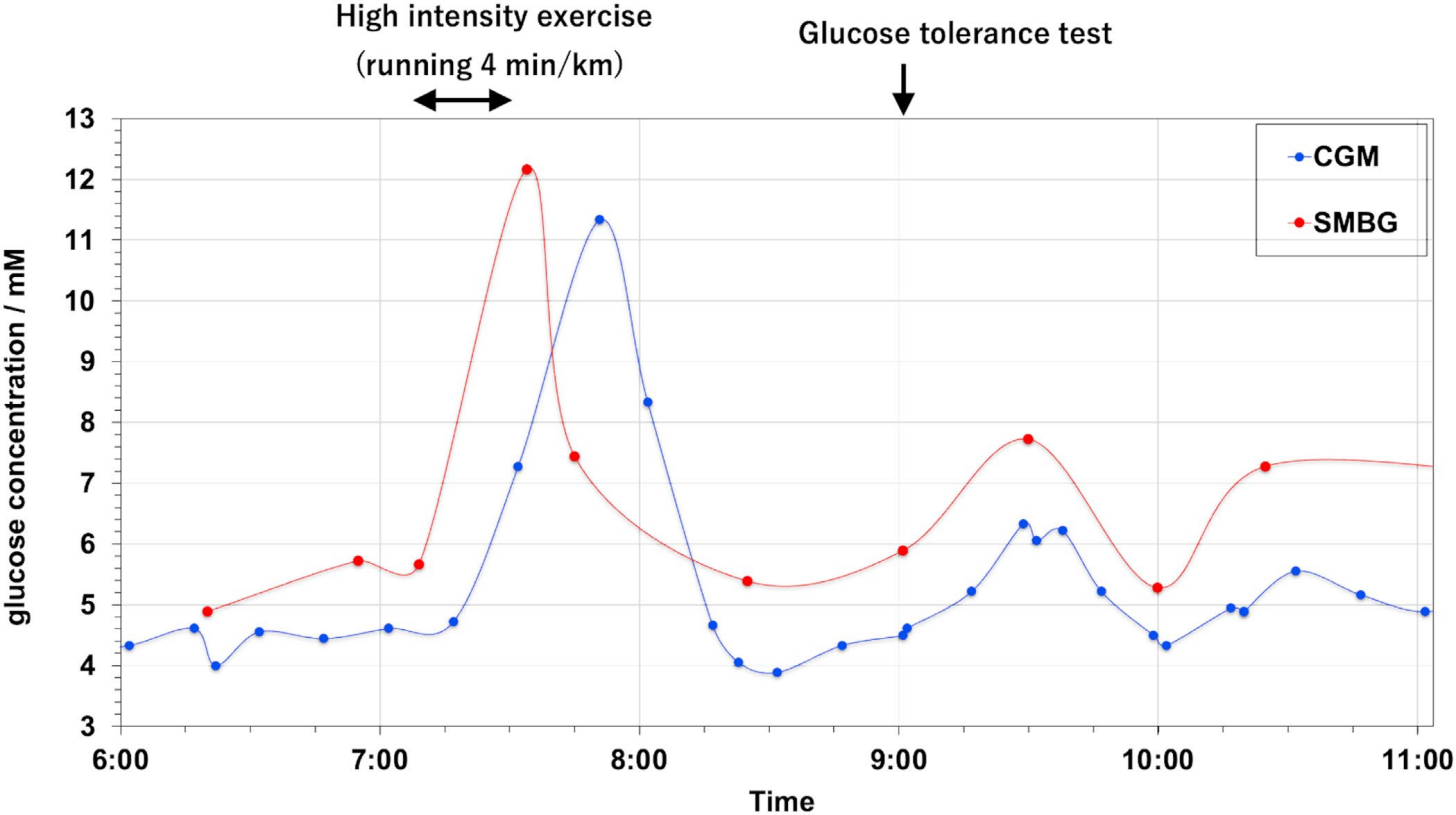
Time course graph of glucose level fluctuation by self‐monitoring blood glucose (SMBG) and continuous glucose monitoring (CGM) during high‐intensity running and glucose tolerance test after 12 h of fasting (Red: SMBG, Blue: CGM).

We evaluated SMBG‐CGM discrepancies during high‐intensity exercise (sub‐elite runner, 40s, HbA1c 5.1%, fasting glucose 5.4 mM). After 12‐h fasting, the runner ran 5 km at 4 min/km pace, reaching a maximum heart rate of 175 bpm. Maximum glucose recorded was 12.2 mM by SMBG and 11.3 mM by CGM, with CGM showing an approximately 15‐min delay and an approximately 1.5 mM lower average than SMBG (Figure 1), consistent with prior SMBG‐CGM agreement and lag reports (Rebrin & Steil, 2000; Zaharieva et al., 2019). Following exercise, an OGTT was performed, and glucose peaked at 7.7 mM (SMBG) and 6.3 mM (CGM) at approximately 30 min, returning to normal within 1 h.

**FIGURE 2:**
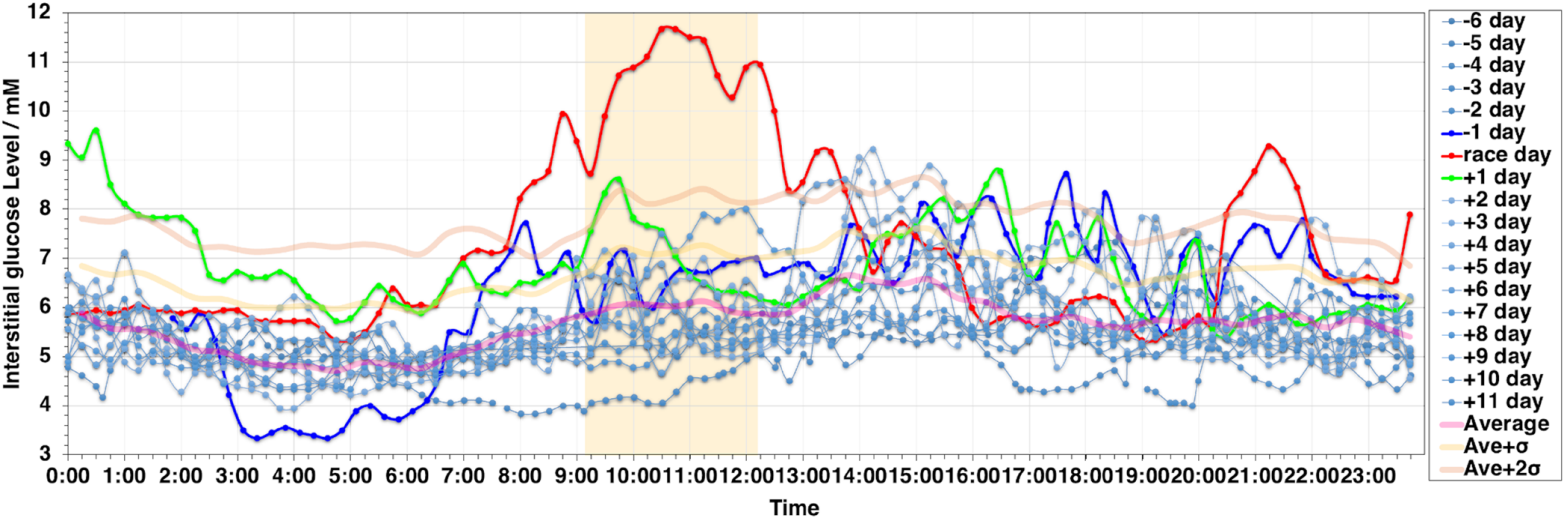
Interstitial glucose concentration plots of 1 sub‐elite runner before and after a full marathon race (Blue: 1 day before the race, Red: Race day, Green: 1 day after the race). Average, average plus a standard deviation, and average plus two standard deviations are indicated as pink, yellow, and skin color line, respectively.

CGM monitored the runner for 18 days around a marathon (Figure 2). CGM data showed glucose rising sharply to 10 mM just before race start, consistently exceeding 10 mM during the race with a peak of 11.7 mM mid‐race, and remaining above 7 mM up to 4 h post‐race. On the night after the marathon, elevated nocturnal glucose levels between 5.7 and 9.6 mM were observed. Comparing CGM data during the race (average pace: 4:14 min/km) and during lower‐intensity marathon practice runs (average pace: 6:16 min/km) demonstrated higher glucose levels during the competitive race. Practice sessions showed CGM glucose mostly within 5–6 mM with an average of 5.4 ± 1.1 mM (Figure S1). These findings indicate that competitive environments induce distinct glucose patterns, with higher and more prolonged hyperglycemia than in training, likely reflecting stress‐related physiological responses.

### 3.2 Statistical analysis of interstitial glucose levels in highly trained long‐distance runners during races

**FIGURE 3:**
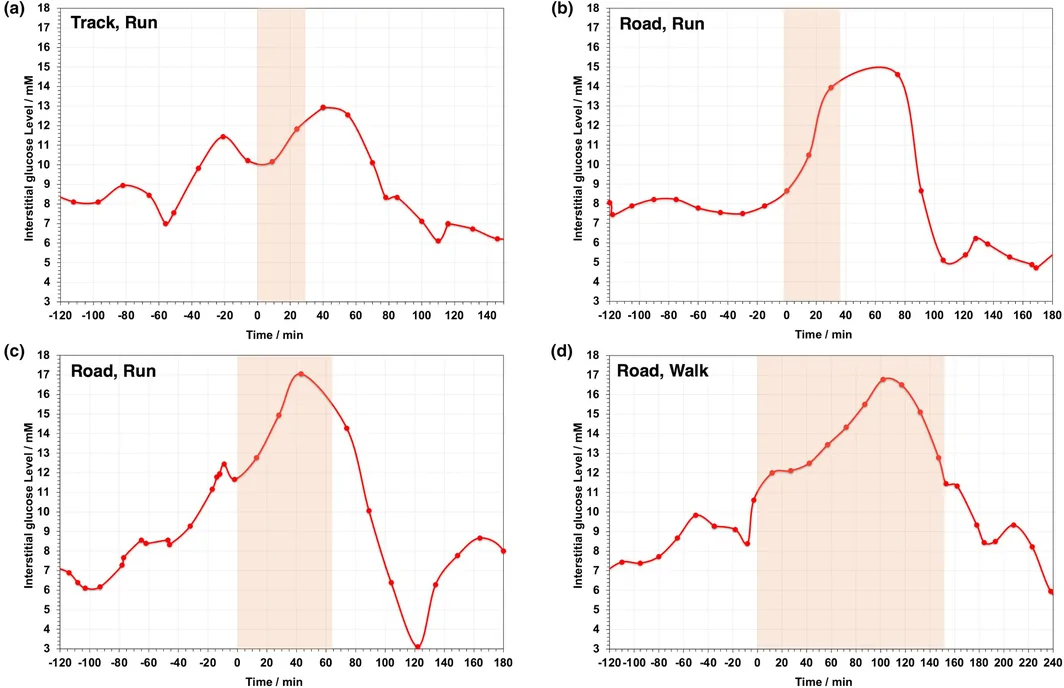
Changes in interstitial fluid glucose concentration from highly trained collegiate long‐distance athletes before/during/after race competitions of different disciplines, shown as individual examples (a: Track running; b: Road relay; c: Road half‐marathon; d: Road race walk). Event distances and finishing placements are not given for these individual panels because the cohort was drawn from a single university squad in a single season, so that distance combined with performance information would allow individual athletes to be identified from publicly available competition records. The pink band indicates the running time with the start time as 0 min.

**FIGURE 4:**
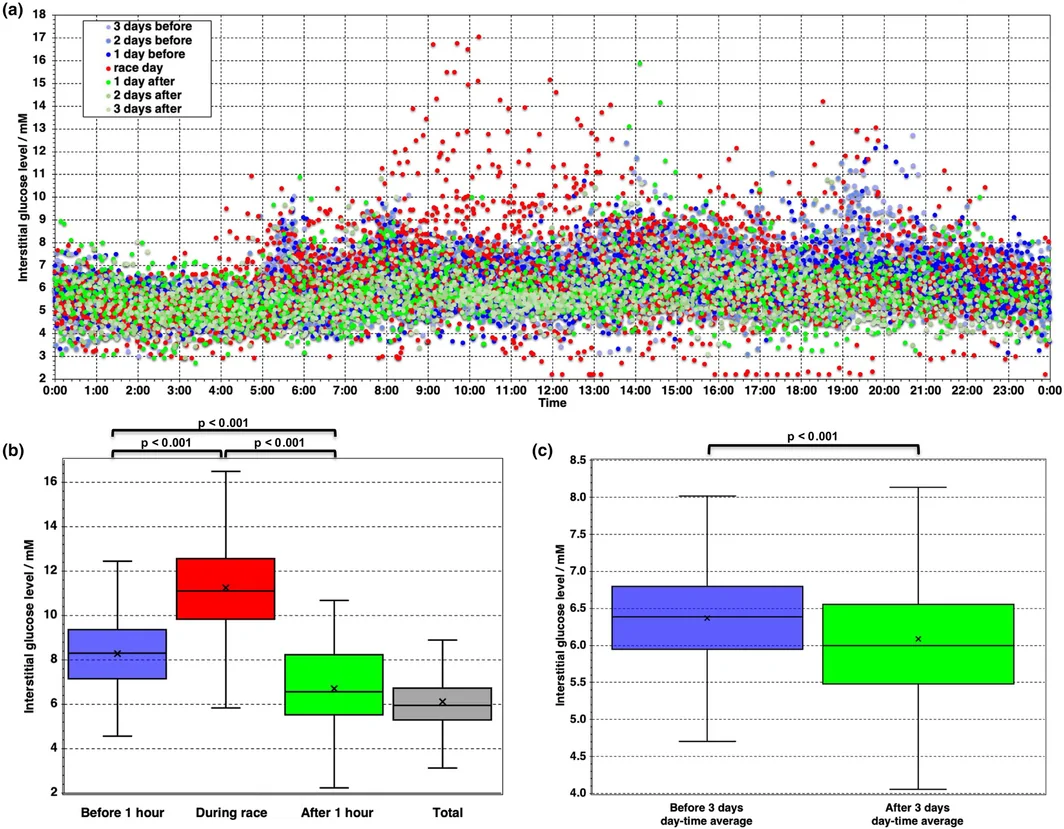
Statistical results from highly trained collegiate long‐distance athletes during real competitions. (a) Actual data plots of interstitial glucose level for 7 consecutive days, including 3 days before and after race day. (b) Statistical analysis of interstitial glucose data 1 h before (blue), during the race (red), 1 h after (green), and total data (gray). (c) Comparison of statistical data 3 days before (blue) and 3 days after the race (green). Post‐hoc paired tests are Holm‐Bonferroni‐adjusted for the three prespecified contrasts (pre vs. during; during vs. post; pre vs. post). Exact *p*‐values are shown numerically above each comparison. Box plots show the median and interquartile range (IQR); whiskers extend to 1.5× IQR, and points beyond are plotted individually as outliers.)

CGM data from 20 athletes during competitions yielded 16,360 points. Table 1 summarizes sample dates, subject numbers, race types, locations, and distances. Figure 3 presents representative CGM data showing mean glucose variations from 2 h before the race to after the finish. While individual pre‐race glucose variability was observed, many athletes showed marked glucose elevation patterns: a sharp rise just before race start and gradual increases during the race. Approximately 90 min before races, glucose increases aligned with supplemental meal intake were also seen. After race completion, glucose levels typically dropped sharply within 1 h. Figure 4a displays individual glucose trajectories spanning 3 days pre‐ and post‐race. Figure 4b shows mean glucose levels segmented into 1 h before, during, and 1 h after the race: overall mean was 6.1 ± 1.3 mM, rising to 8.3 ± 1.5 mM in the pre‐race hour, peaking at 11.2 ± 2.2 mM during races, then declining to 6.7 ± 1.7 mM in the post‐race hour. As shown in Table 2, interstitial glucose increased significantly from the pre‐race hour to competition and decreased from competition to the post‐race hour (mean differences, 95% CIs, and effect sizes are reported in Table 2). The one‐way repeated‐measures ANOVA showed a significant main effect of time (*F*(2,220) = 225.79, *p* < 0.001, partial h2 = 0.67). All post‐hoc paired comparisons are Holm‐Bonferroni‐adjusted; adjusted *p*‐values remained <0.01 for pre versus during and during versus post, and non‐significant for pre versus post where noted; effect sizes and confidence intervals are unchanged.

**TABLE 2:** Paired comparisons of interstitial glucose across time windows with mean differences (mM), 95% confidence intervals, paired *t*‐tests, and within‐subject effect sizes (Cohen's dz. and Hedges' g).

Comparison	Mean differences	95% CI low	95% CI high	*t*	Cohen's dz	Hedges' g (within)
Before 1 h versus during race	3.122	2.718	3.525	15.319	1.454	1.444
During race versus after 1 h	−4.560	−5.078	−4.041	−17.430	−1.654	−1.643
Before 1 h versus after 1 h	−1.438	−1.806	−1.070	−7.749	−0.735	−0.730

*Note*: Before 1 h: The glucose variation of 60 min preceding competition start; After 1 h: The glucose variation of first 60 min after race completion. Two‐sided paired *t*‐tests; *p*‐values adjusted by Holm‐Bonferroni across the three prespecified contrasts (pre vs. during; during vs. post; pre vs. post). Mean difference shown with 95% CI. Effect sizes: Cohen's dz. and Hedges' g (within).

### 3.3 Comparison of competition versus high‐intensity training (within‐subject analysis)

Twenty paired observations from 15 athletes who wore CGM during a sanctioned competition and a high‐intensity training session within 8 days on a matching course type (track to track; road to road) at the same prescribed race pace were analyzed. The external characteristics of each pair are summarized in Table 3. The within‐subject maximum interstitial glucose was higher in competition than training (12.91 ± 1.93 vs. 11.02 ± 1.18 mM), yielding a mean difference of +1.89 mM (95% CI 1.06–2.72); paired *t*(19) = 4.78, *p* = 1.31 × 10^−4^; Cohen's dz. = 1.07; Hedges' g (within) = 1.03. A two‐level repeated‐measures ANOVA (condition: competition vs. training) corroborated this effect (*F*(1,19) = 22.83, *p* < 0.001, partial h2 = 0.55). As a complementary scale‐free metric, the geometric mean ratio (competition/training) was 1.166 (95% CI 1.088–1.249), indicating approximately 17% higher peaks in competition. Collectively, these within‐subject results show higher peak glucose in competition than in training performed at the same prescribed pace, independent of inter‐individual baselines (Figure S2). Two exploratory analyses were performed to characterize the residual uncertainty arising from the absence of internal‐load measurement; neither is intended to resolve causal attribution. First, we examined whether the competition‐training difference varied across events spanning a wide range of event distances and externally characterized exercise demands. Grouped by competition distance, the mean difference was +2.17 mM (SD 0.99; *n* = 4) for events under 10 km, +2.15 mM (SD 1.28; *n* = 9) for 10–15 km, and +1.40 mM (SD 2.62; *n* = 7) for events longer than 15 km; among running events the difference was inversely, but not significantly, related to competition distance (Pearson *r* = −0.41, *t*(16) = −1.80, *p* = 0.09; Spearman rho = −0.45). The two race‐walk pairs showed a larger mean difference (+4.00 mM) than the 18 running pairs (+1.66 mM, SD 1.60). We note that an account in which competition elicited a higher internal exercise intensity than training would predict the largest differences in the longer events, where the scope for competition to exceed habitual training intensity is greatest; the observed gradient runs in the opposite direction. Given the sample size and the exploratory nature of this comparison, we regard this as a pattern that is not aligned with that account rather than as evidence against it. Second, we asked how large an unmeasured competition‐training difference in exercise intensity would need to be to account for the observed +1.89 mM. Using competition pace recovered from official published results for the running competitions (*n* = 16, spanning a pace range of approximately 40 s/km), peak competition glucose showed no detectable association with competition pace (slope + 0.011 mM per s/km, 95% CI −0.099 to +0.121; *t*(14) = 0.22). Because the point estimate is indistinguishable from zero, it cannot itself bound the required difference; taking instead the upper limit of the confidence interval, a competition‐training pace difference of approximately 16 s/km would be required, corresponding to a deviation of about 9% from the prescribed race pace. This calculation is a sensitivity exercise only: the slope is estimated from between‐athlete and between‐event variation rather than from within‐pair contrasts, the pace actually achieved in training was not recorded so within‐pair pace differences cannot be computed, and pace is in any case only a proxy for internal load, which is additionally influenced by terrain, ambient conditions, hydration status, and fatigue. In a sensitivity analysis restricted to running events only (*n* = 18; excluding the two race‐walk pairs), the competition‐training peak‐glucose difference was +1.66 mM (95% CI 0.86–2.46; paired t(17) = 4.40, *p* < 0.001; Cohen's dz. = 1.04; geometric mean ratio 1.147), compared with +1.89 mM (geometric mean ratio 1.166) in the full cohort. The two race‐walk pairs therefore contributed to, but did not account for, the overall difference. The magnitude of the pre‐race glucose rise (peak minus the pre‐race resting baseline) was positively correlated with the peak glucose reached during competition (Pearson *r* = 0.44, *p* = 0.010; *n* = 33 race events with paired pre‐race and during‐race CGM windows), indicating that athletes with larger anticipatory pre‐race elevations also reached higher glucose during competition. For pre‐race versus post‐race baselines, glucose was 6.4 ± 0.7 mM pre‐race versus 6.1 ± 1.1 mM post‐race, a small but significant 0.3 mM elevation (*p* < 0.001) (Figure 4c).

**TABLE 3:** External characteristics of the 20 competition‐training pairs.

Pair	Discipline	Course type	Competition distance (band)^a^	Competition pace (band, min/km)^a^
1	Running	Track	10–15 km	<=2:55
2	Running	Track	<10 km	<=2:55
3	Running	Track	<10 km	<=2:55
4	Running	Track	<10 km	<=2:55
5	Running	Track	10–15 km	<=2:55
6	Running	Road (relay)	<10 km	<=2:55
7	Running	Road (relay)	10–15 km	<=2:55
8	Running	Road (relay)	10–15 km	<=2:55
9	Running	Road (relay)	>15 km	<=2:55
10	Running	Road (relay)	10–15 km	2:55–3:05
11	Running	Road (relay)	>15 km	2:55–3:05
12	Running	Road (relay)	>15 km	2:55–3:05
13	Running	Road (relay)	>15 km	2:55–3:05
14	Running	Road	>15 km	2:55–3:05
15	Running	Road (relay)	10–15 km	>3:05
16	Running	Road (relay)	>15 km	>3:05
17	Running	Track	10–15 km	Not available
18	Running	Cross country	10–15 km	Not available
19	Race walking	Track	10–15 km	4:00–4:15
20	Race walking	Road	>15 km	4:00–4:15

*Note*: Each competition was paired with a high‐intensity training session prescribed at the same race pace, on a matching course type, within 8 days. Competition pace was recovered from official published results and is reported in bands; it was not available for two pairs. The pace actually achieved in the training sessions was not recorded, so the degree of matching attained within each pair cannot be quantified. Two of the road‐relay competitions involved a substantial net ascent or descent; the remainder were flat or undulating. Exact distances, finishing times, placings and event identities are not reported, and pair order does not correspond to that of any other table, because the cohort was drawn from a single university squad in a single season and these details would permit individual athletes to be identified from publicly available competition records. Internal load (heart rate, power, blood lactate) was not measured in either condition.

^a^ Bands are used in place of exact values for the reason given above.

## 4 DISCUSSION

This study used real‐world CGM data to characterize glucose dynamics during sanctioned competitive events in highly trained collegiate endurance athletes. The principal observation is a reproducible competition‐specific hyperglycemic response: a sharp pre‐race elevation, sustained intra‐race hyperglycemia (peak approximately 11 mM, with individual maxima reaching 12–13 mM), and rapid post‐race normalization. Critically, within‐subject paired analyses demonstrated that peak glucose during competition exceeded that during training by approximately 1.89 mM (geometric mean ratio 1.166), with a large effect size (Cohen's dz. = 1.08). Because internal exercise load was not measured, this observation cannot be attributed to the competitive context rather than to a difference in internal exercise intensity between the two conditions. Pace was prescribed rather than measured, and neither heart rate, power, nor blood lactate was recorded. The external characteristics that were documented—prescribed pace, distance, and course type (track or road)—constrain mechanical work, but they do not establish equivalence of internal physiological load, which is determined by the individual's response to that work and not by the work alone.

### 4.1 Interpretation in the context of stress physiology

**FIGURE 5:**
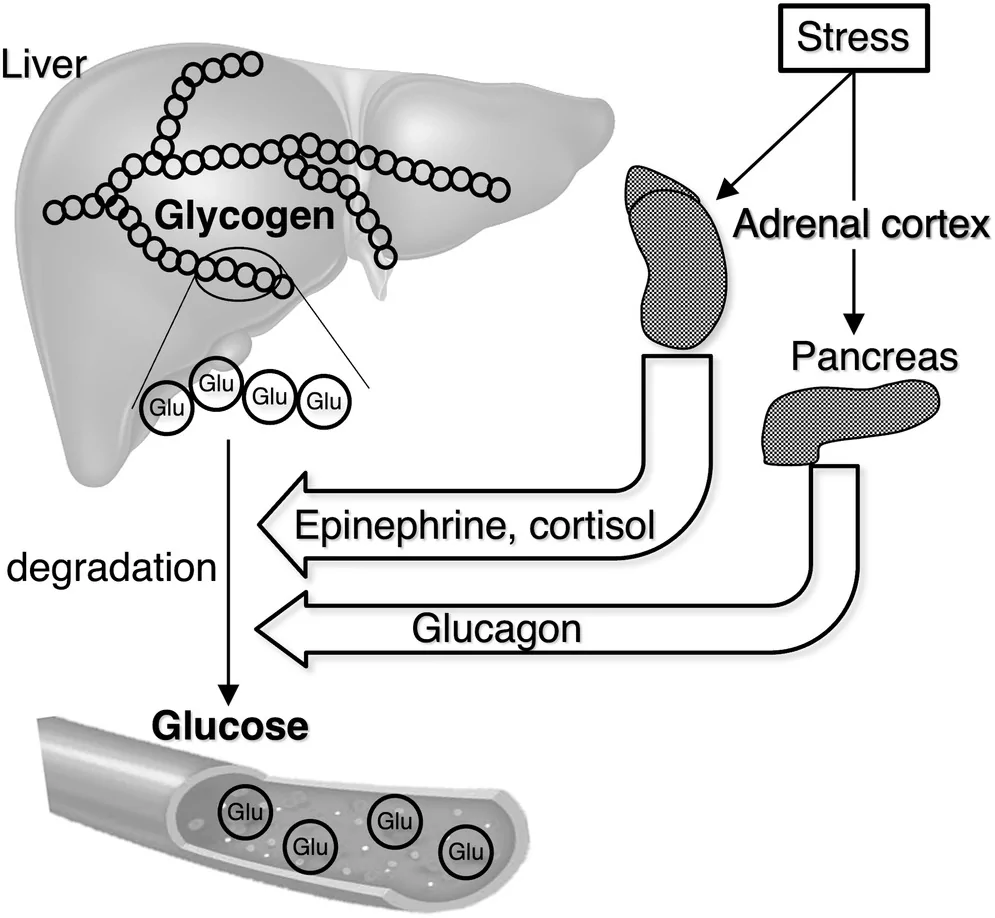
Proposed mechanism of blood glucose elevation by anti‐stress hormones secreted in response to stress factors such as tension and excitement.

Elevated glucose during exercise has been reported (Holzer et al., 2022), and increased epinephrine correlates with higher glucose during exercise (Howlett et al., 1999). In adrenalectomised humans, exogenous adrenaline infusion during exercise directly increases hepatic glucose production, providing causal evidence that adrenergic signaling drives exercise hyperglycemia (Howlett et al., 1999). Notably, the marked hepatic glucose output of high‐intensity exercise is itself predominantly catecholamine‐mediated feed‐forward and is preserved even under exogenous glucose infusion (Marliss & Vranic, 2002); intensity‐driven and stress‐driven hyperglycemia therefore converge on a common adrenergic hepatic pathway rather than representing competing mechanisms. Our preliminary fasted‐state experiment showed glucose elevation during and after intense running detected by both SMBG and CGM, suggesting that liver glycogen breakdown may drive glucose elevation under conditions where exogenous fuel is absent (Figure 5). Established literature indicates that glucagon and adrenaline increase when the brain senses glucose decline, promoting hyperglycemia (Adeva‐Andany et al., 2018; Hamilton et al., 2018; Jiang & Zhang, 2003; Sanders & Ritter, 2000). Our main‐cohort data showed glucose above 10 mM before race start, rising to 11.1 mM during the race—a pattern that contrasts with prior reports where moderate exercise lowers glucose (Afsheen Syeda et al., 2023). The preliminary SMBG‐CGM comparison involved one participant and served solely for feasibility verification; all primary findings are based on the main cohort of 20 athletes with systematic within‐subject analyses.

Field CGM case studies in ultra‐endurance events have described glucose profiles alongside in‐race carbohydrate refueling practices (Sengoku et al., 2015). In our cohort, however, solid carbohydrate intake was completed by > = 120 min pre‐start and carbohydrate‐containing fluids were prohibited during races; thus, the observed competition‐phase glucose elevations are unlikely to be explained by in‐race carbohydrate ingestion. Because the events studied here were high‐intensity but short (<2 h), post‐race glucose normalized rapidly, in contrast to ultra‐endurance or overtraining paradigms in which post‐exercise elevations can persist (Flockhart & Larsen, 2024).

Twenty athletes showed distinct glucose patterns. Figure 3 shows individual examples of real‐time glucose trends across disciplines and course types; these representative individual trajectories are not intended to infer a relationship with performance outcomes. Analysis revealed pre‐race glucose elevation (approximately 1 h before start) over 2 mM above baseline derived from 3 days before/after the race (Figure 4c). This elevation is unlikely to be explained solely by pre‐race supplements taken 1.5–2 h before the race, as average glucose rise from supplements did not exceed 8 mM (Figure 4a). During races, glucose peaked at 11.1 mM, nearly double normal resting values. While high‐intensity training showed similar patterns, race‐day glucose levels were higher (Figure S2). Multiple‐comparison control was implemented via Holm‐Bonferroni, and all inferences were unchanged under classical Bonferroni, supporting the robustness of the findings. This anticipatory elevation is already present at rest, roughly 1 h before the start and before the warm‐up. Warm‐up routines were described by the athletes as comparable on competition and training days, but they were not recorded, and pre‐race intake on the day was not documented at the level of individual food items. Differences in warm‐up or in acute pre‐race intake therefore cannot be excluded as contributors to the pre‐exercise divergence.

Physiological interpretation requires caution. The liver stores approximately 100 g of glycogen, while muscles store approximately 500 g (Hargreaves & Spriet, 2020; Taylor et al., 1996; Wasserman, 2009), but muscle glycogen cannot release glucose into the blood (Ramnanan et al., 2011). Elevated glucose during races is attributable to endogenous (hepatic) glycogen mobilization because in‐race carbohydrate‐containing fluids were prohibited by event regulations and solid carbohydrate was completed > = 120 min before the start. The pre‐race glucose elevation extending up to 3 days before the race suggests increased glucose turnover during the preparation period. In our dataset, pre‐start glucose often exceeded approximately 10 mM and reached higher levels during competition, suggesting substantial glucose mobilization in the competitive context. This interpretation is discussed cautiously in relation to established hepatic glucose output physiology while recognizing that causality cannot be established without direct hormonal measurements. In healthy individuals, clinically significant hypoglycemia during exercise is generally prevented by counter‐regulatory increases in hepatic glucose production (glycogenolysis and gluconeogenesis), although circulating glucose may decline during prolonged exercise (Ahlborg et al., 1974; Wasserman, 2009). Therefore, an acute performance collapse is unlikely to be explained by liver glycogen depletion alone. Consistent with this, no athlete in our cohort exhibited intra‐race hypoglycemia despite the high glucose turnover.

### 4.2 Practical applications

Our results suggest practical implications. First, consistently elevated pre‐competition glucose levels indicate the need to tailor pre‐race nutrition strategies; individualized glucose monitoring may guide adjustments to standard carbohydrate‐loading, as athletes with high pre‐race glucose may not require aggressive carbohydrate supplementation. Second, glucose levels during competition were markedly higher than during matched‐intensity training, highlighting the unique metabolic demands of competitive environments; coaches may implement training simulations mimicking competition stress to foster metabolic adaptations; such simulations are intended to elicit endogenous neuroendocrine and hepatic glucose responses that exogenous carbohydrate intake does not reproduce, and to acclimate athletes to the competition‐specific metabolic milieu. Third, post‐race glucose dropped sharply within 1 h, suggesting a window for optimized recovery nutrition; prompt intake could aid in faster recovery by replenishing glycogen stores. Overall, efficient liver glycogen utilization is critical for sustaining performance during competition. Both oral carbohydrate strategies and psychological preparation to manage stress responses should be personalized based on individual CGM data.

### 4.3 Limitations

Several important limitations must be acknowledged. First and most critically, internal physiological load was not measured. Heart rate, power output, and blood lactate were not recorded in either condition, and the pace actually achieved in each session was not documented; pace was prescribed (R pace from current VDOT tables) rather than measured. Internal load is the individual's psychophysiological response to a given external workload and is therefore not fixed by prescribing pace. Consequently, we cannot determine whether the higher glucose observed in competition reflects the competitive context or a difference in internal exercise intensity between the two conditions, and we make no causal attribution. Second, we correct a statement made in earlier versions of this manuscript: psychological state was not entirely unmeasured. A pre‐competition questionnaire was administered at several events and included a single bipolar item on pre‐race affect, anchored from “extremely tense” through “calm and neutral” to “maximally aroused”. In an exploratory analysis restricted to the one competition for which each athlete's individual exercise window could be reconstructed, this item showed no statistically significant association with the pre‐competition glucose rise under any of four pre‐race window definitions (*n* = 7–8 per analysis). We report this for completeness and do not regard it as supporting any interpretation. The measure cannot test the hypothesis, for four reasons: (i) the competition was a relay, so the hour preceding an athlete's own start does not represent a comparable physiological state across athletes who ran the earliest and the latest legs, and who differed in waiting time, feeding, and circadian position; (ii) a single bipolar item collapses tension and positive arousal, which are not neuroendocrinologically equivalent, onto one axis; (iii) an athlete whose tension rises abruptly just before the start and one whose arousal builds over several hours generate different temporal patterns that a single fixed analysis window cannot distinguish; and (iv) self‐report collected within a squad is subject to social‐desirability bias. Validated instruments (Competitive State Anxiety Inventory‐2 [CSAI‐2], State–Trait Anxiety Inventory [STAI]) together with direct measurement of stress hormones (plasma or salivary epinephrine, norepinephrine, cortisol, glucagon) are what this question requires; none of these was available here. Accordingly, although the observed glucose elevations during competition are consistent with stress‐mediated hepatic glucose output as described in classical exercise physiology literature (Hems et al., 1978; Hems & Whitton, 1980; Howlett et al., 1999; Sherwin & Sacca, 1984), we cannot establish causality, and the patterns may reflect multiple interacting factors including psychological arousal, anticipatory anxiety, sympathetic nervous system activation, and the metabolic demands of competitive performance. Third, performing repeated finger‐stick SMBG sampling on all athletes during sanctioned competitions was not feasible or ethical, as it would (i) disrupt performance and interfere with competition rules and logistics, (ii) pose safety and handling concerns in high‐intensity, crowded settings, and (iii) exceed institutional approvals for on‐course data collection. Fourth, although competition‐training pairs were matched by course type and within an 8‐day window, complete elevation metrics were not uniformly recorded for road sessions, leaving some residual environmental variability. In one pair the CGM record ended while interstitial glucose was still rising during the competition, so the competition peak for that pair is a lower bound. Moreover, because the pace actually achieved in each session was not recorded, the degree of external‐workload matching attained within each pair cannot be quantified; matching was by prescription, and the tolerance actually achieved is unknown. Fifth, our cohort comprised only male collegiate athletes (Tier 3: Highly Trained/National), so generalization to female athletes, masters athletes, or higher‐tier (Tier 4–5) elite athletes warrants caution. Finally, focusing solely on glucose overlooks other key factors in exercise metabolism; lactate re‐metabolism, for example, may affect glucose dynamics during competition (Messonnier et al., 2021; Yang et al., 2020). Real‐time interstitial glucose remains the only biomarker practically measurable during races, underscoring the need for additional continuous monitoring technologies.

### 4.4 Future research directions

Future studies should integrate CGM with: (a) validated psychometric assessments (CSAI‐2, STAI) synchronized to pre‐, during‐, and post‐competition phases; (b) heart rate variability (HRV) analysis as a non‐invasive autonomic nervous system index; and (c) salivary or plasma catecholamine and cortisol measurements timed to CGM windows. Such multi‐modal approaches would allow direct testing of the stress‐glucose hypothesis and clarification of individual differences in stress responsiveness. Coupling CGM with heart rate and VO2max data could yield comprehensive insights, and the development of continuous lactate monitoring would complement glucose data, enabling a more complete metabolic assessment. Lastly, correlating glucose patterns with performance outcomes may help identify optimal metabolic profiles for peak results.

## 5 CONCLUSION

Continuous glucose monitoring revealed consistent elevations in interstitial glucose before and during competitive events in highly trained athletes, with peaks approximately 17% higher than in high‐intensity training performed at the same prescribed race pace (geometric mean ratio 1.166; mean difference +1.89 mM, 95% CI 1.06–2.72). Because internal exercise load was not measured in either condition, these findings are descriptive and do not establish that the difference arises from the competitive context rather than from a difference in internal exercise intensity. They provide a field reference for glucose behavior around competition and a basis for hypothesis‐driven studies in which internal load is measured directly.

## AUTHOR CONTRIBUTIONS

**Taira Kajisa:** Conceptualization; data curation; formal analysis; funding acquisition; investigation; methodology; project administration; software; validation; visualization; writing – original draft; writing – review and editing. **Toshiyuki Sakai:** Conceptualization; investigation; methodology; resources; supervision; writing – original draft; writing – review and editing.

## FUNDING INFORMATION

The work was supported by Japan Keirin Autorace Foundation (2021M‐145), and supplemented by HAKUJU INSTITUTE FOR HEALTH SCIENCE Co., Ltd. and Nisshinbo Holdings Inc. for joint research funding.

## CONFLICT OF INTEREST STATEMENT

T.K. is the Chief Executive Officer and a shareholder of SympaFit Inc. (Tokyo, Japan), a company that develops wearable‐based athlete‐monitoring technologies, including continuous glucose monitoring (CGM)‐based applications. SympaFit Inc. was established after the data for the present study had been collected. The CGM devices used in this study were commercially available products (FreeStyle Libre 2; Abbott Diabetes Care) and were not manufactured by SympaFit Inc. SympaFit Inc. had no role in the study design, data collection, analysis, interpretation, manuscript preparation, or the decision to submit for publication, and provided no funding for this study. The other author (T.S.) declares no competing interests.

## ETHICS STATEMENT

All procedures were conducted in accordance with the Declaration of Helsinki and were approved by the Ethics Review Committee for Medical Research Involving Human Subjects at Toyo University (TU2021‐006). All participants provided written informed consent prior to participation.

## Supporting information


**Figure S1.** Interstitial glucose concentration curves of the sub‐elite runner during a full marathon race and practice (black line: race day, dotted line: practice day). The yellow band indicates the running time of full marathon.

**Figure S2.** Comparison of glucose levels in a long‐distance elite athlete between race and high‐intensity practices (Red: race, Blue and Green: high‐intensity practice).

## Data Availability

All anonymized data and analysis code that support the findings of this study will be made openly available at 10.6084/m9.figshare.22807130.
